# Mass Drug Administration and beyond: how can we strengthen health systems to deliver complex interventions to eliminate neglected tropical diseases?

**DOI:** 10.1186/1753-6561-9-S10-S7

**Published:** 2015-12-18

**Authors:** Eleanor E Macpherson, Emily R Adams, Moses J Bockarie, T Deirdre Hollingsworth, Louise A Kelly-Hope, Mike Lehane, Vanja Kovacic, Robert A Harrison, Mark JI Paine, Lisa J Reimer, Stephen J Torr

**Affiliations:** 1 Liverpool School of Tropical Medicine, Liverpool, UK; 2 Warwick Medical School, Warwick University, Warwick, UK

## Abstract

Achieving the 2020 goals for Neglected Tropical Diseases (NTDs) requires scale-up of Mass Drug Administration (MDA) which will require long-term commitment of national and global financing partners, strengthening national capacity and, at the community level, systems to monitor and evaluate activities and impact.

For some settings and diseases, MDA is not appropriate and alternative interventions are required. Operational research is necessary to identify how existing MDA networks can deliver this more complex range of interventions equitably.

The final stages of the different global programmes to eliminate NTDs require eliminating foci of transmission which are likely to persist in complex and remote rural settings. Operational research is required to identify how current tools and practices might be adapted to locate and eliminate these hard-to-reach foci.

Chronic disabilities caused by NTDs will persist after transmission of pathogens ceases. Development and delivery of sustainable services to reduce the NTD-related disability is an urgent public health priority.

LSTM and its partners are world leaders in developing and delivering interventions to control vector-borne NTDs and malaria, particularly in hard-to-reach settings in Africa. Our experience, partnerships and research capacity allows us to serve as a hub for developing, supporting, monitoring and evaluating global programmes to eliminate NTDs.

## Contribution of LSTM to the control of Neglected Tropical Diseases

Since its foundation, LSTM has played an important role in global efforts against neglected tropical diseases (NTDs). Commencing with Joseph Everret Dutton's pioneering work on trypanosomiasis in 1901, the School now host's the Centre for Neglected Tropical Diseases (CNTD), a key partner in global efforts against lymphatic filariasis (LF), and the A·WOL Consortium, developing novel treatment regimes for prevention of transmission and alleviation of morbidity for onchocerciasis and LF. Other major programmes of research at LSTM currently include human African trypanosomiasis (HAT), visceral leishmaniasis, schistosomiasis, snakebite and dengue. All our work is conducted in partnership with researchers from Africa, the Americas, Asia and Europe.

The School's work extends from basic research into the biology of parasites, vectors, their hosts and snake venoms through applied research to develop new drugs, diagnostics and methods of control through to assisting national control programmes. At each of these levels, there are synergies between NTDs as well as other diseases: methods and systems used to study or control one disease can impact on others. Crucially, ensuring that investment in health system strengthening needed for NTDs will be highly relevant to other conditions in need of health system strengthening to improve health. These synergies extend beyond NTDs, especially to malaria, another important area of health research and delivery at LSTM.

Recognising and exploiting these synergies relies on collaboration between national programmes, communities, international organisations, clinicians, scientists, private companies and policy makers. LSTM's history, partnerships and programmes means that it is uniquely well placed to serve as a nexus to support a truly integrated global programme to eliminate NTDs.

## Where are we?

Many of the global programmes working against NTDs currently rely largely on mass drug administration (MDA) of preventive chemotherapies (e.g., schistosomiasis, onchocerciasis, lymphatic filariasis, soil-transmitted helminths) and active detection and treatment of cases (leishmaniasis, Gambian sleeping sickness). With the notable exception of antivenom to treat snakebite, drugs are currently donated by the pharmaceutical industry, and in the case of MDA programmes distributed to local communities via national public health supply chains and volunteer community drug distributors.

The scale of the MDA programmes is exemplified by the Global Programme to Eliminate Lymphatic Filariasis (GPELF) which delivered a combination of albendazole with either ivermection or diethylcarbamazine citrate to 539 million people in 53 countries in 2011[1]. Despite these enormous achievements of this global programme against LF progress is fragmented[2,3]. On the one hand, nine countries no longer require MDA and 12 have moved to post-MDA surveillance. On the other hand, 17 of the 34 endemic countries in Africa have yet to start[4].

## Achieving the 2020 goals

### Priorities for MDA-diseases

To meet the 2020 goals, operational and implementation research is needed to optimise the scale up of cost-effective and sustainable strategies, especially in fragile and post-conflict countries where health systems are often weak and overburdened (see CAHRD paper HS HR FCAS). Priorities include (i) securing long-term commitment of national and global financing partners, (ii) improving the supply chain of medicines, (iii) strengthening national capacity to implement MDA at the community level and (iv) establishing national systems to monitor and evaluate the activities and impact of national programmes[4].

### Priorities for Non-MDA diseases

MDA will not be adequate for a variety of diseases and settings[5-7]. New tools and strategies are being developed to address these limitations (see CAHRD paper NTD Tools). These include use of (i) Doxycycline in place of ivermectin in areas where onchocerciasis and loiasis are coendemic[7], (ii) delivery and development of affordable and safe antivenoms with region-wide efficacy[8], (iii) interventions to address the social determinants of health including improved sanitation to enhance efforts against schistosomiasis and soil-transmitted helminths, (iv) rapid diagnostic tests (RDTs) and orally-delivered drugs (for stage 2 treatment) to improve the ability of health centres to diagnose and treat human African trypanosomiasis and (v) environmental interventions and vector control to benefit groups that drug-based strategies cannot reach.

### Role of vector control

Adding vector control to the current drug-based methods will not only complement current approaches but accelerate overall progress against NTDs[9]. First, attacking the vector and parasite simultaneously reduces the overall reproductive number. Second, several methods of vector control act against multiple vectors: bednets and indoor residual spraying impact on indoor-biting mosquitoes, triatomine bugs and sandfly, the vectors of malaria, LF and Chagas disease; treatment of livestock with insecticides impacts on tsetse flies, the vectors of sleeping sickness, as well as livestock-biting species of mosquito and sandfly.

### Strengthening health systems

Third, improvements in the capacity of a health centre to diagnose and treat one disease may improve its overall ability to detect and treat other diseases. Against these benefits, provision of services for the treatment and control of NTDs requires dedication of resources and delivering a more complex range of interventions may be challenging for often overburdened health systems to deliver. Adding vector control to the services delivered by, say, a district-level health authority may be particularly difficult, especially where novel methods are being promoted, e.g. use of baits to attract and kill vectors rather than bednets or house spraying. How can health systems be strengthened to deliver complex interventions?

Recent research undertaken by the CNTD suggests a way forward[10]. Njelesani et al. (2014) developed a set of monitoring and evaluation tools to determine the capacity of laboratory systems by analysing, institutions, national governments and international agencies[10] (see CAHRD paper HS Capacity). This approach highlights the strengths and limitations of not only a particular laboratory system but how its function is influenced by the wider enabling environment. This analytical approach can be broadened to evaluate and strengthen national systems (laboratories, vector control departments, mapping departments) that are essential components of an integrated NTD control programme.

The shift from MDA to a more integrated approach, combining interventions against the parasite and vector, means that programmes to eliminate NTDs and malaria are likely to be similar. Indeed, vector control methods used against mosquito-borne diseases such as malaria, LF and dengue (i.e., insecticide-treated bednets, indoor-residual spraying, larval source management) may be identical. Closer collaborations between institutions working to control NTDs and malaria will facilitate sharing of experiences and resources and allow us to realise emerging synergies between interventions directed against NTDs and malaria, especially for disease such as LF in Africa which is largely transmitted by the same vector species[11-13].

## Beyond 2020

Leading up to 2020, the different global programmes working against NTDs will need to comprise a wider range of interventions applied over a greater area, particularly in sub-Saharan Africa. Assuming that efforts to increase funding, capacity and implementation are successful, then the global burden disease of NTDs will decline and with this the scale of MDA. However, two areas will assume increasing importance: management of disability and eliminating the last bastions of NTD.

### Management of disability

Millions of people live with chronic debilitating, disabling, and disfiguring conditions as a consequence of NTD infection[14,15]. Once they become infected or envenomed, there is often little that can be done to reverse this debility. Global commitments have highlighted the importance of alleviating suffering and providing support for those infected with NTDs[1]. However, there has been significantly less progress made establishing clinical and social programmes to provide this support. For example in 2008, despite an estimated 40 million individuals living with LF, only 29 of the 82 endemic countries covered by GPELF at the time reported that they had commenced morbidity management programmes[16].

Even if NTD elimination targets are achieved, millions of women, men and their carers will continue to require clinical and social support including snakebite victims suffering the chronic, typically irreversible, physically and psychologically disabling effects of envenoming. Developing and implementing effective and sustainable models of services to reduce the impacts of the physical and mental disabilities caused by NTDs is an urgent public health priority. There will also be important lessons and synergies from non-communicable diseases such as asthma (see CAHRD paper LH Cough). Considering ways to integrate services including morbidity management programmes into existing community-based approaches will be important when delivering any new services (from drugs, vector control, diagnostics or social support) within affected community. CNTD has recently piloted new innovative community-based tool using mobile phone technology for collecting and mapping cases of LF. Preliminary results are excellent and the work will be expanded across their 12 focus countries and include additional aspects of health information and service delivery.

### Eliminating hard-to-reach foci

Progress towards the elimination of NTDs will be patchy. For instance, progress will be slower in areas affected by conflict or geographical features (e.g., mountainous regions, extensive swamps, dense forest) that hamper delivery of interventions[17,18]. Persistence of disease in these regions will pose two important threats to efforts against NTDs.

First, residual foci can act as a source of parasites that can spill into areas from which the disease has been eliminated; this risk is especially serious for vector-borne diseases and zoonoses where vectors and reservoir hosts are mobile. Second, the persistence of diseases foci can contribute to institutional, funding and community fatigue, especially as elimination is expensive and/or and the disease burden is small relative to other health priorities – snakebite being a particularly pertinent example[19]. Hence the challenge facing NTD endemic countries in the 2020s will shift from scaling up standard interventions to delivering a complex suite of interventions, which may include chemotherapy, environmental management, vector control in hard-to-reach places[17].

The delivery of these interventions will require participation and engagement by communities affected by these diseases, and continued donor and partnership support. Spatial modelling of bednets in DRC found significantly low intervention coverage in rural remote areas, with minimal access to main cities and transport networks[17]. This study highlights the important gaps in coverage and the geographical factors driving them. Similar studies would assist efforts against other NTDs.

It is important that the difficulties of the ‘last mile’ are anticipated and solutions established before persistent foci lead to either re-emergence of disease or fatigue. There are three elements to tackling this problem: (i) rapid identification and monitoring of persistent or re-emerging foci, (ii) delivery of interventions to contain and ultimately eliminate the foci and (iii) maintaining the support of communities, governments and global partners. The requisite tools are already in use or will be available by 2020[9,13]. Here we outline research concerned with delivering strategies to identify and eliminate foci in hard-to-reach settings.

### Identifying foci

Current research is producing a rich source of data and tools to map diseases, vectors, environmental and demographic factors[17,18,20]. These tools can provide the basis of early-warning systems to identify sites where disease may persist and hence guide monitoring activities. Such an early-warning system could be operated by an international programme[21,22] working in partnership with national health systems, making best use of new remote sensing products and practices as they emerge, and in recognition that a focus threatens neighbouring regions and countries[9]. Some risk factors will be spatially and/or temporally stable (e.g. impact of altitude on the bionomics of vectors and parasites), seasonal (e.g., rainfall, temperature) or unpredictable (fragility and conflict). Local communities could play an important role in early warning systems; they will be the first witnesses of resurgences. Research and development will be required to develop robust systems to integrate these data into an overall early warning and establish systems to provide national health systems with appropriate warnings, building on existing global early warning systems for health such as GOARN (Global Outbreak Alert and Response Network) GLEWS (Global Early Warning System).

Guided by a global early warning system, national and local health systems and local communities will provide the basis for monitoring disease. Research will identify how RDTs and innovative monitoring systems (e.g., xenomonitoring vectors and reservoir hosts if appropriate) can be routinely used at sentinel sites, and how data from these can be rapidly disseminated to national and global centres. Information systems between sentinel sites and the early warning system will allow rapid updating of risk assessments[13].

Innovations will all need to be considered in terms of their introduction, acceptability and adherence by those affected communities[23]. They will also need to be considered in terms of how they will be delivered within health systems. Strategies for partnership and engagement with affected communities will need to be developed and tailored to the contexts in which they are delivered.

### Containing and eliminating foci

A flexible and integrated approach will be required to eliminate the last bastions of disease. For example, persistent human African trypanosomiasis foci in West Africa are associated with the difficulties of reaching people living in extensive mangrove swamps[24]. Operational research conducted in these particular settings will be required to identify how communities might be reached.

It seems likely that some foci will persist or re-emerge as a direct result of local economic or socio-political factors. Well-resourced professional response teams could provide a means of containing outbreaks and then provide the basis of a team to re-establish local capacity.

### Maintaining momentum

The endgame is likely to be protracted and maintaining the support and engagement of international partners, governments and communities is crucial. The African Programme for Onchocerciasis Control (APOC) as well as the GPELF use a community-directed strategy for the distribution for MDA, which is vital to the success of both programmes[25-27]. Understanding how to continue to empower and engage communities will be essential to ensure momentum at the community-level is maintained during the endgame. Research using both qualitative (interviews with key informants, focus group discussions with communities) and quantitative (Knowledge Attitudes and Practice surveys) methods with community members and key informants will help inform the best strategies to reach the elimination goals.

## Conclusions

In the lead up to 2020, there is a pressing need to strengthen funding and national capacity to deliver interventions against NTDs, which will be based on, but not limited to, MDA.

In the post 2020 world, the different global programmes working against NTDs will be more focussed on disability management and the identification and elimination of the last bastions of disease.

Systematic analysis of capacity, interventions and impact conducted in the lead up to 2020 will provide the basis for developing strategies to complete ‘the last mile’.

## Competing interests

The authors declare that they have no competing interests.

## Authors contributions

All authors contributed to the development of the final manuscript.

**Figure 1:**
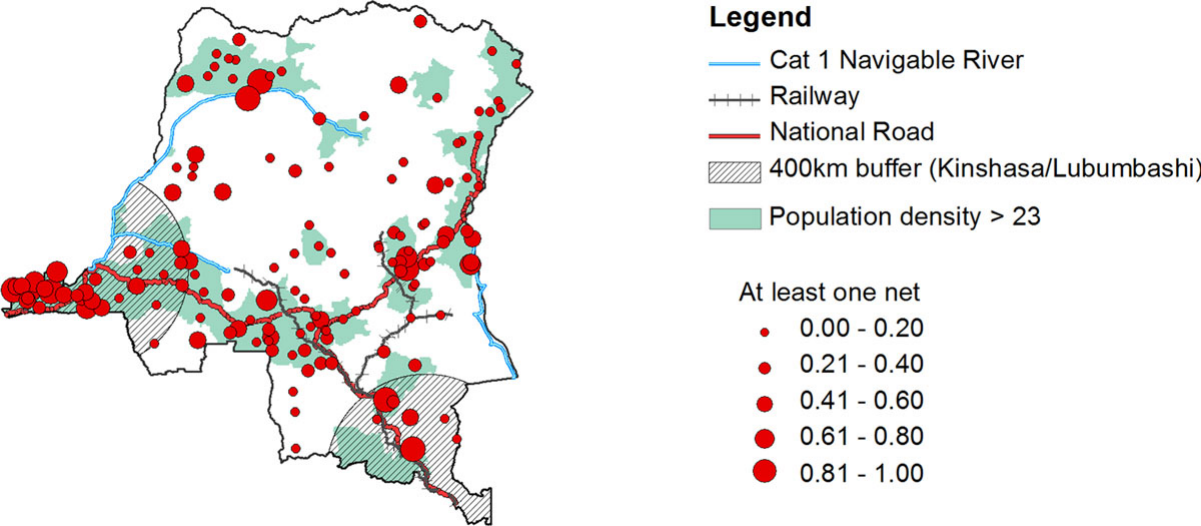
Bed net coverage in DRC.[17]

## Declarations

This article has been published as part of *BMC Proceedings* Volume 9 Supplement 10, 2015: The 2014 CAHRD (Collaboration for Applied Health Research & Delivery) Consultation. The full contents of the supplement are available online at http://www.biomedcentral.com/bmcproc/supplements/9/S10.
